# Genetic Variation and Cerebrospinal Fluid Levels of Mannose Binding Lectin in Pneumococcal Meningitis Patients

**DOI:** 10.1371/journal.pone.0065151

**Published:** 2013-05-31

**Authors:** Matthijs C. Brouwer, Frank Baas, Arie van der Ende, Diederik van de Beek

**Affiliations:** 1 Department of Neurology, Academic Medical Center, Center of Infection and Immunity Amsterdam (CINIMA), Amsterdam, The Netherlands; 2 Department of Genome Analysis, Academic Medical Center, Center of Infection and Immunity Amsterdam (CINIMA), Amsterdam, The Netherlands; 3 Department of Medical Microbiology, Academic Medical Center, Center of Infection and Immunity Amsterdam (CINIMA), Amsterdam, The Netherlands; 4 The Netherlands Reference Laboratory for Bacterial Meningitis, Academic Medical Center, Center of Infection and Immunity Amsterdam (CINIMA), Amsterdam, The Netherlands; University of Padova, Medical School, Italy

## Abstract

It has been suggested that genetic variants in mannose binding lectin (*MBL2*) influence susceptibility and outcome of invasive pneumococcal disease. We assessed the influence of genetic variation in *MBL2* on susceptibility, outcome and causative serotype of pneumococcal meningitis in a prospective nationwide cohort study including 299 white patients and 216 controls. We assessed functionality of the genetic polymorphisms by measuring levels of MBL, C3a, iC3b, C5a and sC5b-9 in cerebrospinal fluid. We also performed a meta-analysis of studies on *MBL2* polymorphisms and susceptibility to invasive pneumococcal disease. The risk of contracting pneumococcal meningitis was substantially increased for white individuals homozygous with the defective *MBL2* 0/0 genotype (odds ratio [OR] 8.21, 95% confidence interval [CI] 1.05–64.1; p = 0.017). CSF MBL levels were significantly lower in patients with the A/0 and 0/0 genotype compared to homozygotes for the wild-type alleles (A/A; p<0.001). CSF MBL levels were positively correlated with C3a and iC3b levels, indicating complement activation by the lectin pathway. The effect of *MBL2* genetic variants on susceptibility remained robust in a meta-analysis including 5 studies with 287 patients (OR 2.33, 99% CI 1.39–3.90). We conclude that *MBL2* polymorphisms influence CSF MBL levels and substantially increase the risk of pneumococcal meningitis.

## Introduction

Bacterial meningitis is a serious and life-threatening disease [1], [2]. *Streptococcus pneumoniae* is the predominant pathogen in adults, causing 70% of all cases, and results in a mortality rate varying from 19% to 37% [1], [3], [4]. Neurological sequelae, including hearing loss [5], focal neurologic deficits [6], and cognitive impairment occur in 30–52% of surviving patients [7], [8]. Susceptibility to invasive pneumococcal disease can be increased due to deficiencies in the immune system, in example after splenectomy, with systemic diseases such as cancer or diabetes, or use of immunosuppressive drugs, but can also occur from genetic defects [6], [9]–[11].

In a systematic review and meta-analysis, genetic polymorphisms in the *MBL2* gene, coding for complement factor mannose binding lectin (MBL), were shown to increase the risk for invasive pneumococcal disease with an odds ratio (OR) of 2.57 (99% confidence interval [CI] 1.38–4.80) [9]. Binding of MBL and other lectins to bacteria constitutes the first step in activation of the lectin activation pathway of the complement system in infectious disease [12]. Subsequent association of MBL with MASP2 results in cleavage of both complement component 4 (C4) and 2 (C2), generating the C2aC4b complex which, by cleaving C3, activates the final common complement activation pathway [12]. Three polymorphisms (rs1800450, rs5030737 and rs1800451) in the first exon of *MBL2* result in three variant structural alleles (also referred to as variant B for rare allele at rs1800450, variant C for rare allele at rs1800451 and variant D for rare allele at rs5030737), which are associated with decreased MBL concentrations and thereby decreased activation of the complement system [13]. Patients with two variants alleles (either variant B, C, or D – together referred to as 0/0) have an increased risk for pneumococcal infections in particular caused by low virulent genotypes [14]. Another *MBL2* polymorphism in the promoter region (−221G/C; rs7096206), usually described as the X/Y variant, has been show to negatively influence transcription and result in lower MBL levels as well. This polymorphism has however not been associated with an increased risk of pneumococcal infection. Genetic MBL deficiency has also been associated with disease severity and outcome in pneumococcal infections [15], [16].

We performed a prospective nationwide genetic association study in patients with community-acquired pneumococcal meningitis to investigate the effect of the structural variants of *MBL2* in susceptibility, severity, and outcome. By analyzing clinical data and cerebrospinal fluid (CSF), we examined the impact of *MBL2* variants on complement activation.

## Methods

In the nationwide prospective cohort study we included bacterial meningitis patients older than 16 years of age with positive cerebrospinal fluid (CSF) cultures who were identified by the Netherlands Reference Laboratory for Bacterial Meningitis (NRLBM, Academic Medical Center, Amsterdam) from March 2006 to June 2009. Methods have been described previously [17]. The treating physician was contacted, and informed consent was obtained from all participating patients or their legally authorized representatives. Patients with hospital-acquired bacterial meningitis and negative CSF cultures were excluded. To guarantee similar exposure to bacteria and socio-economic background of patients and controls, we selected patients’ partners or their non-related proxies living in the same dwelling as controls for exposure/susceptibility [9], [18]. Controls were excluded if related to the patient. Data on age, sex and ethnicity of patients and controls were collected. Blood from patients and controls for DNA extraction was collected in sodium/EDTA and DNA was isolated with Gentra Puregene chemistry (Qiagen). The region including the functional *MBL2* polymorphisms rs5030737 (Arg52Cys, variant B), rs1800450 (Gly54Asp, variant C) and rs1800451 (Gly57Glu, variant D) was fully sequenced by Sanger sequencing. The promoter SNP rs7096206 was genotyped using a TaqMan SNP Genotyping Assay (Applied Biosystems).

A previously reported power analysis showed that 295 patients would be needed to confirm the association found for susceptibility to pneumococcal disease of the exon 1 variants in the meta-analysis, with 80% power using a p-value of <0.05 [9]. We calculated whether the genotype frequencies in white controls concurred with the Hardy-Weinberg equilibrium (HWE) by use of a χ^2^ and exact test with one degree of freedom with a p<0.05 to indicate significance. The primary analysis for susceptibility to pneumococcal meningitis was performed in white patients, and a subgroup analysis was performed for immunocompetent white patients. As our study is a validation of the role of *MBL2* SNPs in invasive pneumococcal disease, we used a significance level of p<0.05. We updated the meta-analysis of the role of *MBL2* SNPs in susceptibility to invasive pneumococcal disease by adding the results of the current study and other recent studies. Studies were selected that provided genotype data for *MBL2* genotypes for white patients with invasive pneumococcal disease confirmed by culture of PCR from blood, joint fluid or CSF, and controls. The Mantel-Haenszel fixed-effects model was used to calculate the OR and 99% CI [9]. Heterogeneity of study results was assessed with the i^2^ statistics, in which i^2^>50% was used to indicate significant heterogeneity.

We further analyzed the influence of the *MBL2* SNPs on disease severity, mortality and rate of unfavorable outcome, determined by the score on the Glasgow Outcome Scale. This is a well-validated instrument with good inter-observer agreement. A score of 1 on this scale indicates death; a score of 2 indicates a vegetative state; a score of 3 indicates severe disability; a score of 4 indicates moderate disability; and a score of 5 indicates mild or no disability. A favorable outcome was defined as a score of 5, and an unfavorable outcome was defined as a score of 1 to 4.

CSF of patients was obtained with the diagnostic lumbar puncture. Subsequently, CSF and WBC were stored separately at −80°C. Controls were patients evaluated for acute headache, in whom a subarachnoid hemorrhage was excluded as cause of their headache by CSF examination. CSF findings were within normal limits in all controls. Leftover CSF was collected, spun down and supernatant was stored at −80°C until analysis. CSF complement component MBL levels were determined using the Hycult ELISA kits HK323 according to the protocol of the manufacturer. CSF complement component C3a, iC3b, C5a and terminal complement complex (TCC; sC5b-9) levels were determined using the Microvue C3a, iC3b, C5a and sC5b-9 Quidel ELISA kits according to the manufacturer’s instructions. Strength of relationships between MBL, C3a, iC3b, C5a and sC5b-9 levels was assessed by Spearman’s correlation tests.

The Mann-Whitney U test and Kruskal-Wallis 1-way ANOVA were used to identify differences in baseline characteristics between groups with respect to continuous variables, and dichotomous variables were compared with use of the χ^2^ test. These statistical tests were two-tailed, and a p-value of <0.05 was regarded as significant. Differences in genotype frequencies were analyzed with the χ^2^ or Fishers’ exact tests by use of SPSS19.

The medical ethics committee of the Academic Medical Center, University of Amsterdam, Amsterdam, the Netherlands, approved the study and informed consent procedure. Written informed consent was obtained from all participating patients or their legally authorized representatives.

## Results

From March 2006 to June 2009, a total of 468 episodes of community-acquired CSF culture-proven pneumococcal meningitis were included. DNA samples were obtained from 301 white patients with pneumococcal meningitis (64%) and 216 controls. Common reasons for not collecting DNA were: patient already deceased, blood withdrawal for DNA isolation not performed, no informed consent, and transfer of the patient between wards or hospitals. Sex, age and ethnicity of patients and controls were similar (Table 1). The median age of the patients included in the genetic analysis was 60 years and 46% were male. Predisposing conditions for meningitis were identified in 66% of patients, and 23% were immunocompromised (Table 2). A total of 25 (8%) patients died and 85 (28%) had and unfavorable outcome defined as a score of 1–4 on the Glasgow Outcome Scale.

**Table 1:** Baseline characteristics of pneumococcal meningitis patients and controls.

Characteristic	Patients with DNA (n = 312)	Controls (n = 227)	p-value^a^
Age (median- IQR^b^)	59.9 (46.0–68.1)	58.6 (46.3–66.4)	0.50
Male sex	143 (46%)	108 (48%)	0.69
Ethnicity			
White	299	216	0.92
African	9	10	0.35
Asian	4	1	0.40

a Mann-Whitney U or Chi^2^, ^b^IQR – interquartile range.

**Table 2:** Clinical characteristics 299 white patients with pneumococcal meningitis on admission and outcome.

Characteristics	No./no. of patients^a^
Median age, yr	60 (45–68)
Male sex	138 (46%)
Duration of symptoms <24 hours	140/291 (28%)
Predisposing conditions	197/297 (66%)
Otitis media/sinusitis	145/297 (49%)
Pneumonia	28/286 (10%)
Immunocompromised state^b^	69/298 (23%)
Symptoms on presentation	
Headache	231/267 (87%)
Nausea	165/260 (63%)
Neck stiffness	224/289 (78%)
Temperature ≥38°C	245/297 (81%)
Signs of septic shock^c^	79/293 (27%)
Score on Glascow Coma Scale	10 (8–13)
Altered mental status (Glascow Coma Scale <14)	244/298 (82%)
Coma (Glascow Coma Scale <8 )	44/298 (15%)
Systemic complications^d^	108/298 (35%)
Neurologic complications^e^	185/295 (63%)
Outcome	
Unfavourable	85/298 (28%)
Death	25/298 (9%)

a Data are number/number evaluated (%), and median (interquartile range).

b Immunocompromised was defined as the use of immunosuppressive drugs, the presence of diabetes mellitus or alcoholism, and human immunodeficiency virus (HIV) infection. ^c^Defined as a systolic blood pressure ≤90 mmHg, a diastolic blood pressure <60 mmHg and/or heart rate ≥120/min. ^d^Systemic complications were defined as septic shock (diasystolic blood pressure <60 mmHg), respiratory failure and need for mechanical ventilation during admission. ^e^Neurologic complications were defined as an altered mental status, focal neurologic deficits, hydrocephalus, seizures, cerebrovascular events (infarction, hemorrhage, cerebral venous sinus thrombosis), cerebral abscesses or empyema’s during admission.

Genotyping was successful in 515 of 517 (99.6%) samples (299 patients and 216 controls) and the genotype frequency of controls was in concordance with the HWE for all 4 SNPs (rs5030737 [B], P = 0.30; rs1800450 [C], P = 0.65; rs1800451 [D], P = 0.95; rs7096206 [XY], P = 0.98). Genotype frequencies of 299 white patients with pneumococcal meningitis and 216 controls are present in table 3. The risk of contracting pneumococcal meningitis was substantially increased for white individuals homozygous with the 0/0 genotype (odds ratio [OR] 8.21, 95% confidence interval [CI] 1.05–64.1, p = 0.017). None of the individual SNPs showed a significant association with susceptibility to pneumococcal meningitis, although a trend towards the increased susceptibility was identified for individuals homozygous for the variant allele of the Gly54Asp SNP (p = 0.059, Table 3). In the subgroup analysis for immunocompetent white patients, 7 patients were homozygous for variant alleles compared to 1 control (OR 6.84, 95% CI 0.83–56.1; p = 0.069). The XY promoter SNP was not associated with susceptibility overall and in the subgroup analyses. When comparing the genotypes previously associated with MBL deficiency (XA/0 and 0/0) with all other genotypes, no association with susceptibility was identified.

**Table 3:** Distribution of *MBL2* polymorphisms in white patients with pneumococcal meningitis and controls.

Polymorphism	Allele/genotype patients					Allele/genotype controls					Recessive model	
	A	0	AA	A0	00	A	0	AA	A0	00	p-value	OR (95% CI)
Arg52Cys (B)	552	46	256	40	3	391	41	175	41	0	0.140	–
Gly54Asp (C)	525	73	234	57	8	388	44	173	42	1	0.059	5.91 (0.73–47.61)
Gly57Glu (D)	586	12	287	12	0	423	9	207	9	0	–	–
B,C and/or D (0)	473	125	185	103	11	343	89	128	87	1	0.017	8.21 (1.05–64.09)
	Y	X	YY	XY	XX	Y	X	YY	XY	XX	Dominant model	
−221G/C (YX)	453	154	171	111	17	335	95	131	73	11	0.40	–
	XA/0+0/0		Other genotypes			XA/0+0/0		Other genotypes				
Deficient genotypes	46		252			29		186			0.54	–

A = common allele, 0 = variant allele, OR = Odds ratio, CI = confidence interval.

*MBL2* genotype was not related to disease severity: the rate of septic shock on admission, systemic complications and unfavorable outcome were similar for all *MBL2* XY and exon 1 genotypes. None of the 11 patients with the 0/0 genotype died compared to 25 of 285 patients (9%) with A/A or A/0 genotypes, but this difference was not statistically significant (p = 0.61).

Pneumococcal serotype was available for 272 patients: serotype 3 in 31 patients (12%), 7F in 29 (11%), 23F in 23 (9%), 22F in 20 (8%), and other serotypes in 169 patients (62%; Table S1). Serotype was available for 9 patients with the *MBL2* 0/0 genotype: serotype 6B in two, and 7F, 8, 15B, 17F, 19F, 23F, and 35F each in one patient. Seven of 9 patients with this genotype (78%) were infected by a serotypes associated with low virulence (attack rates <10 cases of invasive pneumococcal disease/100.000), [19] as compared to 160 of 260 patients (62%) with a A/0 or A/A *MBL2* genotype (p = 0.32). Twenty-six of 37 patients (70%) with deficient genotypes XA/0 and 0/0 were infected with serotypes associated with low virulence compared to 130 of 218 patients (60%) with sufficient *MBL2* genotypes (p = 0.22).

**Figure 1:**
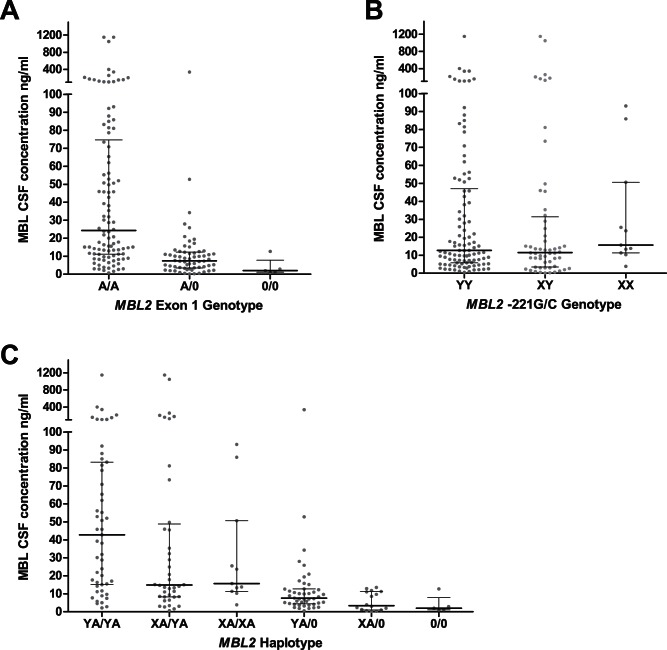
MBL levels in CSF per *MBL2* genotype.

CSF of patients was obtained from the diagnostic lumbar puncture from 155 pneumococcal meningitis patients. CSF MBL levels were significantly higher in pneumococcal meningitis patients compared to negative controls (26 patients in whom lumbar puncture was performed to rule our subarachnoid hemorrhage with normal CSF; median 13.6 ng/ml [IQR 7.1–51.6] vs. 3.1 [IQR 2.2–4.0], p<0.001). Patients homozygous for the wild-type MBL genotype had significantly higher CSF MBL levels compared to heterozygotes and patients homozygous for variant alleles (A/A median 23.8 ng/ml [IQR 11.1–72.8], A/0 7.4 ng/ml [IQR 3.3–12.5], 0/0 2.24 ng/ml [IQR 1.1–5.4], p<0.001 Kruskal-Wallis test; Figure 1A). The XY SNP did not significantly influence CSF MBL levels (Figure 1B); no influence of the XY SNP was observed in a subgroup analysis of patients with the A/0 or A/A genotypes. However, combining the exon 1 and XY variants into 6 haplotypes showed a significant overall difference in CSF MBL levels between groups (Figure 1C). CSF MBL levels were higher in patients with systemic complications, unfavorable outcome and those who died, although this difference was not statistically significant (p = 0.053 for death, p = 0.085 for unfavorable outcome; Table 4). In a correlation analysis of CSF levels of MBL and C3a, iC3b, C5a and sC5b-9 we identified a positive correlation between MBL and C3a (ro 0.42, p<0.001) and iC3b (ro 0.37, p<0.001) CSF levels. No correlation was identified between CSF MBL levels and C5a or sC5b-9. SNPs in *MBL2* did not influence levels complement factors C3a, iC3b, C5a and sC5b-9.

**Table 4:** Association of CSF MBL levels in pneumococcal meningitis patients with complications during admission and outcome.^a^

	Present	Absent	p-value^b^
Systemic complications	16.1 (7.3–77.1)	11.3 (6.4–31.2)	0.079
Neurologic complications	14.5 (6.2–66.0)	11.7 (7.4–34.8)	0.540
Unfavourable outcome	19.6 (7.6–75.4)	12.2 (6.4–36.8)	0.085
Death	23.9 (9.9–166.4)	13.1 (6.5–42.3)	0.053

a Presented values are median levels in ng/ml (interquartile range).

b Determined with the Mann-Whitney U test.

**Figure 2:**
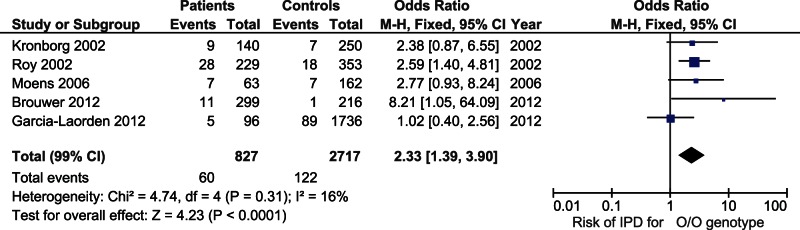
Forest plot of studies on *MBL2* polymorphisms and invasive pneumococcal disease.

In a literature search we identified 9 studies assessing the effect of *MBL2* SNPs on invasive pneumococcal disease (for search strategy see Supporting Material S1) [14], [16], [20]–[25]. Three studies were performed in pneumococcal pneumonia patients, and were only included if the *MBL2* genotype frequencies of bacteremic patients was specified. Two studies were performed in the same population and the smaller of the two was therefore excluded from the meta-analysis [16], [24]. Two additional studies were excluded since ethnic background of the entire study population was African [14], or since all patients were HIV-infected [25]. Ethnicity of included patients was white in 4 of 5 included studies, and was predominantly white (98%) in the remaining study. A total of 827 patients and 2717 controls were included in the meta-analysis (Figure 2). The frequency of the 0/0 genotype between studies was highly variable and ranged from 3.7–12.2% in patients and 0.5–5.1% in controls. The 0/0 genotype was associated with an increased susceptibility to invasive pneumococcal disease (OR 2.33, 99% CI 1.39–3.90, p<0.001). There was no significant heterogeneity between studies (i^2^ = 16%).

## Discussion

Patients homozygous for variant alleles in exon 1 of *MBL2* had 8-fold increased susceptibility to pneumococcal meningitis. *MBL2* variants resulted in lower CSF levels of MBL, and CSF levels of MBL were associated with systemic complications and poor disease outcome. The risk for pneumococcal meningitis that we identified to be associated with these variant alleles is substantially higher than the previously assumed for invasive pneumococcal disease [20]–[22], [24]. The overall OR for invasive pneumococcal disease associated with *MBL2* variants in our meta-analysis was 2.3 (99% CI 1.39–3.90, p<0.001).

Carriers of the X allele of the -221GC promoter polymorphism in our study were not at increased risk for pneumococcal meningitis. This is in line with previous studies on genetic variation in *MBL2* and pneumococcal disease that showed no association of the XY SNP with susceptibility [20], [22], [24]. As the combination of the X allele with the A/0 genotype has been described to cause decreased levels of MBL similar to those seen in 0/0 patients, the absence of an effect of the XY polymorphism may however also be explained by a lack of power to detect an association [15], [26], [27].

CSF MBL levels were higher in patients with increased disease severity and poor disease outcome. High serum MBL levels have previously been associated with favorable disease outcome in invasive pneumococcal disease [15]. This inverse relation between disease phenotypes (meningitis *vs.* bacteremia) for MBL levels and outcome can be explained by the role of complement activation and enhanced inflammatory response specifically in the central nervous system, which is associated with neurological complications and poor prognosis [17]. An interesting observation was that all pneumococcal meningitis patients with the *MBL2* 0/0 genotype survived, as compared to 9% mortality among patients with the A/A and A/0 genotype. Our findings suggest the lectin pathway as a target for adjunctive therapy in pneumococcal meningitis. Antibodies to MASP-2 have shown to block lectin pathway driven inflammation and limit tissue loss in ischemic pathologies [28].

CSF MBL levels were positively correlated to C3a and iC3b levels, but did not influence levels of C5a and sC5b-9. The correlation between CSF MBL with C3a and iC3b levels implies that the lectin pathway is important in pneumococcal disease and influences initial complement activation. This is in contrast with an experimental study of complement deficient mice that showed that only a small proportion of C3 binding to pneumococci (4%) was MBL dependent [29]. A recent study showed that MBL does not bind to *S. pneumoniae in vitro*, and other lectins (ficolin-2 and collectin-1) were responsible for binding of the pneumococcus and activation of the lectin pathway [30]. Furthermore, serum from MBL deficient persons did not result in a decrease in C3b deposition on pneumococci. The mechanism by which MBL influences susceptibility and disease severity in pneumococcal disease remains therefore unclear. As a direct interaction of MBL with *S. pneumoniae* was not found, the effect on pneumococcal disease is probably indirect [30].

Our study has limitations. First, differences in inclusion criteria and choice of controls between studies included in the meta-analysis may limit its value. The difference in genotype *MBL2* frequencies between studies included in the meta-analysis is a serious point of concern, especially since the frequency of patient homozygous for exon 1 variant alleles was very low in our study. The genotype frequency found in our control population was however identical to that reported in the HAPMAP database for the Northern and Western European population, and is therefore likely to be a representative sample. Our choice of controls (patients’ partners or proxies) ensured similar socioeconomic background, age, sex and most importantly, exposure to bacteria. Furthermore, the nationwide design reduces the risk of stratification bias. Another limitation is that the sample size is relatively small for a genetic association study. However, this sample size was derived after a careful power-calculation based on three previous genetic association studies on *MBL2* genotype in pneumococcal disease and is therefore adequate [9]. Validation in other larger populations of pneumococcal meningitis and invasive pneumococcal disease patients is still warranted, as it will add to the strength of the evidence found so far. Larger studies are needed to further study the effect of *MBL2* SNPs on outcome and pneumococcal serotype. As we show that low CSF MBL levels are associated with improved outcome, larger studies may find that patients with deficient *MBL2* genotypes have a better prognosis of pneumococcal meningitis. Finally, an association of the XY SNP with susceptibility that may have be missed due to a lack of power in our and previous studies may be identified in a larger cohort.

A large number of studies have been performed on MBL function, deficiency and serum levels, but no unequivocal conclusion can be drawn on its importance in infectious and other diseases [31]. Our study provides the first data on MBL deficiency in pneumococcal meningitis, and shows MBL deficiency caused by genetic variants in *MBL2* exon 1 to be associated with a substantial increase in susceptibility to pneumococcal meningitis. Phase 1 and 2 trials of MBL replacement therapy have been performed in small selected populations [31]–[33]. MBL reconstitution in MBL deficient individuals to prevent pneumococcal meningitis is currently however still a theoretical option. Our data suggest that blocking the lectin pathway might be an interesting strategy in pneumococcal meningitis.

## Supporting Information

Table S1 Serotypes identified in 272 patients with pneumococcal meningitis.
(DOC)

Supporting Material S1 Search strategy.
(DOC)
