# Supplementary material for: Genetic Variation and Cerebrospinal Fluid Levels of Mannose Binding Lectin in Pneumococcal Meningitis Patients
Source: PLoS One. 2013 May 31;8(5):e65151. doi: 10.1371/journal.pone.0065151 (PMC3669246; doi:10.1371/journal.pone.0065151)
Supplement: Table S1 — Serotypes identified in 272 patients with pneumococcal meningitis. (DOC) [file pone.0065151.s001.doc]

**Supporting Table S1**

Serotypes identified in 272 patients with pneumococcal meningitis

| Serotype | Frequency (%) | Serotype | Frequency (%) |
| --- | --- | --- | --- |
| 1 | 11 (3.7) | 16F | 3 (1.0%) |
| 3 | 32 (10.7) | 17F | 1 (0.3%) |
| 4 | 15 (5.0%) | 18B | 3 (1.0%) |
| 6A | 5 (1.7%) | 18C | 2 (0.7%) |
| 6B | 16 (5.4%) | 19A | 2 (0.7%) |
| 7F | 29 (9.7%) | 19F | 14 (4.7%) |
| 8 | 19 (6.4%) | 20 | 1 (0.3%) |
| 9N | 2 (0.7%) | 22F | 20 (6.7%) |
| 9V | 6 (2.0%) | 23A | 2 (0.7%) |
| 10A | 10 (3.4%) | 23B | 5 (1.7%) |
| 11A | 6 (2.0%) | 23F | 23 (7.7%) |
| 12F | 3 (1.0%) | 24F | 4 (1.3%) |
| 14 | 5 (1.7%) | 31 | 4 (1.3% |
| 15A | 1 (0.3%) | 33F | 6 (2.0%) |
| 15B | 6 (2.0%) | 35B | 1 (0.3%) |
| 15C | 2 (0.7%) | 35F | 4 (1.3%) |
