# Supplementary material for: Genetic Variation and Cerebrospinal Fluid Levels of Mannose Binding Lectin in Pneumococcal Meningitis Patients
Source: PLoS One. 2013 May 31;8(5):e65151. doi: 10.1371/journal.pone.0065151 (PMC3669246; doi:10.1371/journal.pone.0065151)
Supplement: Supporting Material S1 — Search strategy. (DOC) [file pone.0065151.s002.doc]

**Supporting material S1**

**Search Stragey**

("Mannose-Binding Lectin"[Mesh] OR "Mannose-Binding Lectin" OR MBL2 OR MBL OR "Complement Pathway, Mannose-Binding Lectin"[Mesh]) AND ( "*Streptococcus pneumoniae*"[Mesh] OR "Pneumococcal Infections"[Mesh] OR "Meningitis, Pneumococcal"[Mesh] OR Pneumococc* OR *S. pneumoniae* OR Streptococcus*)
